# Increasing Social Communication by Teaching Texting to Autistic Children

**DOI:** 10.1007/s41252-023-00322-9

**Published:** 2023-03-09

**Authors:** Jenna Gilder, Marjorie H. Charlop

**Affiliations:** 1 Claremont Graduate University, Claremont, CA 91711 USA; 2 Psychological Science Department, Claremont McKenna College, 850 Columbia Avenue, CA 91711 Claremont, USA

**Keywords:** Autism, Text messaging, Texting, Social communication, Intervention, Technology

## Abstract

**Objectives:**

In the present study, we increased the social communication of four autistic children by teaching texting conversation skills on smart phones.

**Methods:**

A multiple baseline design across two dyads was used to assess the texting conversation intervention, with additional generalization probes taken across texting partners and FaceTime® sessions. One-month maintenance probes were also assessed.

**Results:**

All four participants increased their conversational texting, and their conversation content was novel. Generalization across texting partners occurred, and skills were maintained. Appropriate verbal content spoken during FaceTime® probes was also observed.

**Conclusions:**

Results are discussed in terms of the potential benefits of teaching autistic children social communication through text.

Conversational speech, the backbone of social communication, is the back and forth exchange of information between two individuals (Larson & McKinley, 1998). However, this important skill is often a shortcoming in autistic children’s speech (e.g., Marion et al., 2012). Over the past several decades, there has been a whole host of interventions created to teach conversational skills to autistic children including basic prompting and reinforcement techniques (Endicott & Higbee, 2007; Goldsmith et al., 2007; Koegel et al., 2010; Lechago et al., 2010; Williams et al., 2000), cue cards and scripts (Brown et al., 2008; Charlop-Christy & Kelso, 2003; Ganz et al., 2008; Pollard et al., 2012; Reagon & Higbee, 2009), and video modeling (Boudreau & Harvey, 2013; Charlop et al., 2008; Charlop et al., 2010). In recent times, due to a combination of technological innovation and decreased cost of technology (Edwards, 2018), social communication has been occurring, primarily for neurotypical populations, more often on computers and other technological devices, and social relationships have been formed and maintained through the use of digital media (Pew, 2015a, 2018a).

In a study by the Pew Research Center, 71% of neurotypical 13–17-year olds reported being Facebook® users, while 52% used Instagram®, and 41% used Snapchat®. Also, multiplayer videogames represented another popular platform for communication and social engagement (Pew, 2015b). MacMullin et al. (2016) conducted a study comparing responses on a technology use survey completed by parents of neurotypical children and teens with parents of autistic children and teens. The results indicated that parents reported that their autistic children demonstrated a greater interest in Internet and videogame use than parents of neurotypical peers did. This suggests that social communication through technological means could possibly potentiate the development of new friendships and increase social engagement for autistic children as it does with neurotypical children (Scott, 2008).

To date, there is little research using such digital means for social communication with autistic children. Cheng and Ye (2010) and Ke and Moon (2018) used a collaborative virtual reality environment while Gallup et al. (2016) used a massive multiplayer online role-playing game (MMORPG). Both Chung et al. (2015) and Brodhead et al. (2019) used video formats in that they devised a video calling procedure and video game play platform, respectively. Finally, Scott (2008) increased social communication via emails. Although these studies are promising, more research is needed to explore digital and technological advances to use for social communication for autistic children.

One area that has not yet been explored for autistic children is social communication on a cell phone. It is estimated that 95% of adolescents in the USA own a mobile phone and 77% own a smart phone (Pew, 2018b). In addition, the age range of smart phone ownership is expanding, with an increasing number of children as young as eight reporting owning a smart phone Smart phone ownership is common across families of various income levels with 96% of American adults earning less than $30,000 a year reporting ownership of a cell phone and 71% indicated owning a smart phone (Pew, 2018a). Research on early cell phone use has indicated social benefits. It has been found that children and teens equate having a cell phone with a higher social status, and that such possession enabled them to connect with friends and maintain relationships, all while having the perception of independence. Data on cell phone use by autistic youth is scarce (Durkin et al., 2010). It is the cell phone’s texting feature that is preferred to maintain friendships and acquaintances (Pew, 2015b).

Glenwright and Agbayewa (2012) presented a computer application via computer that worked similarly to that of text “bubbles.” The participants were presented with a scenario and then instructed to write a comment to a friend (i.e., a compliment) within the text bubble. The friend, in turn, then had the opportunity to write a response to the comment. This simulation of a brief text-like conversation held promise for a texting intervention.

The present study taught four autistic children social communication within conversational dyads through a text messaging intervention on smart phones. Independent texting conversations were assessed, and generalization of this skill to other texting partners (i.e., parents, siblings) was measured. Conversation content novelty was additionally measured. Ancillary gains across FaceTime® probes for verbal social communication were assessed. Finally, maintenance of texting skills at a 1-month follow-up period was measured.

## Method

### Participants

The participants were four autistic children, between the ages of 8.7 and 10.9 years old, who were all diagnosed according to *The Diagnostic and Statistical Manual of Mental Disorders* (DS APA, American Psychiatric Association, 2013). All participants attended after-school social skill groups. Requirements of each participant were that they needed to be literate and demonstrate the basic motor skills needed to use a mobile phone. Pretests to assess these skills are discussed below.

The participants were grouped into two dyads based on age, interests, social skill group affiliation, and desire to communicate with each other. The dyad pairings, ages of the participants, genders, ethnicities, expressive verbal age (EVT-III), receptive language age (PPVT-IV), and autism severity (CARS-2) are presented in Table 1.

**Table 1:** Participant characteristics

Dyad	Participant	Chronological age in years (sex)	Ethnicity	EVT-III	PPVT-IV	CARS-2
Dyad 1	Bennett	10.9 (Male)	white	9.4	8.5	Mild/moderate
	Milo	8.7 (Male)	white	**	**	Mild/moderate
Dyad 2	Anna	10.2 (Female)	Korean-American	7.5	7.4	Mild/moderate
	Veronica	10.8 (Female)	Latina	**	**	Mild/moderate

#### Dyad 1

Bennett was 10 years and 9 months old at the start of the study (see Table 1). In addition to his autism spectrum disorder (ASD) diagnosis, Bennett also had an ADHD diagnosis. Bennett had difficulty maintaining a back and forth conversation. He usually engaged in monologues about inappropriate topics. Bennett and his texting partner Milo often partnered together in social skill sessions and shared similar interests. Milo, an 8-year, 7-month-old boy, primarily discussed preferred topics and communicated more with therapists as opposed to peers.

#### Dyad 2

When engaging in conversations, Anna (10.2 years) stood too close to her peers, discussed inappropriate topics, focused primarily on her perseverative interests, and had difficulty in engaging in a back and forth conversations. Veronica (10.8 years) showed some rigidity in terms of conversational topics and activities, and had difficulty responding appropriately to social cues. They had both participated in activities with each other inside the social skill group and had indicated an interest in being friends. When they interacted during activities in the social group, they had difficulty maintaining a back and forth conversation.

### Procedure

#### Materials

For the study, one iPhone® version 5 and three Samsung Galaxy phones belonging to the participants’ parents or the participants themselves were used. Additional materials included four guidebooks. The guidebooks focused on content and included a different example of a back and forth texting conversation for two people. Materials also included iPads® used to record the texting training sessions and computers used by the children and the experimenter when conducting sessions over videoconferencing software due to Covid-19 lockdown which occurred during this study. Lastly, the texting intervention was implemented using an application called TextFree®.

#### Baseline

Baseline sessions, intervention training sessions, generalization texting partner probes, and FaceTime® probes were conducted in two different lounge settings (1.5 m by 3 m) at the social skill program that the participants attended weekly. The participants were exposed to these rooms prior to the study. Each participant was assigned to one of the two rooms through the baseline and training sessions. The first lounge style room contained a couch, a table, and a chair. The second room contained three chairs, a circular table, and a bookshelf. Partway through baseline, due to Covid-19 lockdown, the setting was moved to the children’s respective homes. The timing of the change in settings from the social skill program to the home environment corresponded with the restrictions put in place by the government.

#### Design

A non-concurrent multiple baseline design across dyads was used to examine the effect of the texting intervention upon appropriate text exchanges between participants. In addition, response generalization probes across verbal conversations over FaceTime® and stimulus generalization probes across parents and siblings were assessed. Probes for stimulus generalization across texting partners and FaceTime® occurred during baseline, post-intervention, and at maintenance.

#### Pre-baseline Assessments

Prior to taking part in the study, the parents of each participant, as well as the participants themselves, were asked if they wanted to establish a texting relationship with a friend. Next, the participants were asked to read two different sentences presented on a phone in text format to assess their reading ability (i.e., “I like to play with dinosaurs and talk to my friends about them,” “I also have a sister named Chloe who sometimes plays with me”). In addition, the participants were asked to write two different sentences that were presented verbally (i.e., “I like playing sports outside,” “My favorite sport is soccer”).

#### Baseline Probes

During baseline, the dyads were created based on child preference, and each participant was initially separated and seated in two different lounge-style rooms. They were then each given their phone that had been turned on and given the instruction “send a text to your friend _______.” Their peer’s phone number was already pre-programed into the phone and could be accessed by typing in their friend’s name and taping the number when it appeared below the name. Dyads were given 10 min per each baseline session to simulate how much texting would be done during intervention.

#### Text Conversation Intervention

**Fig. 1:**
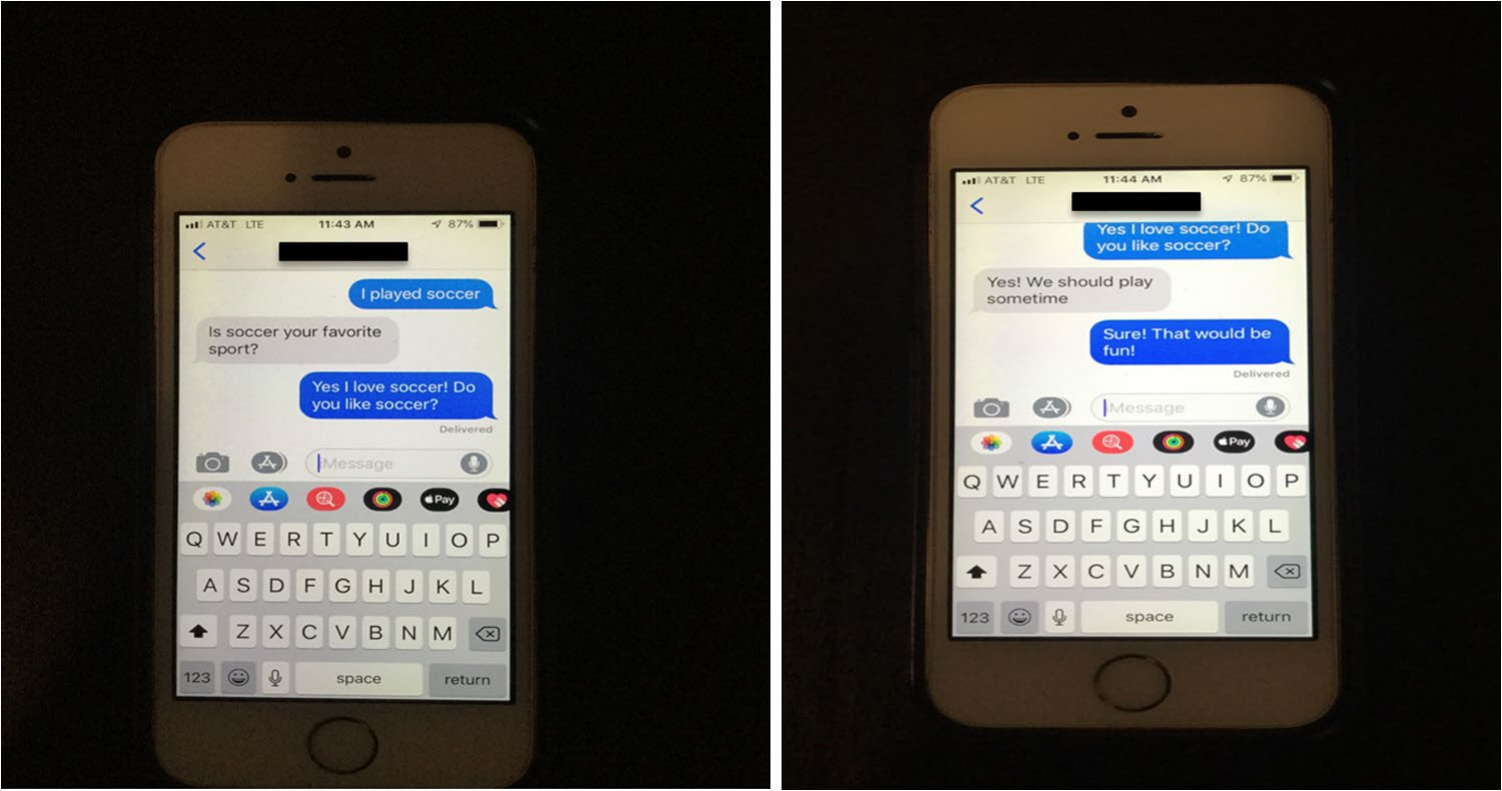
Example of textual conversation between two friends

The intervention involved teaching the participants to have a back and forth conversation through text. Once again, the dyads were separated and were taught this skill using two sample conversations (see Fig. 1). In each of the sample conversations, two fictional persons created by the experimenter were conversing: Conversation A was between Brad and Kim, and conversation B was between Claire and Luis. The conversations were presented using multiple pictures of a smart phone with one to two novel lines of text presented on the screen in each picture. These sample conversations were centered around two different main topics: Conversation A was about soccer, and conversation B was about movies. In addition, these conversations both began and ended with some form of a greeting (i.e., hi, hello, see you later, bye). Both conversations had a central topic, and both of the conversers in each scripted conversation asked and responded to questions. The two sample conversation books were alternated across the participants. That is, the two participants in each dyad never had the same conversation manual during a single session (session one: Veronica had book A, and Anna had book B; session two: Veronica had book B, and Anna had book A). This was done in an effort to encourage more varied speech.

During intervention, both participants were handed one of the conversation guidebooks and were told to read it. The participants were given 5 min at the start of each 10-min intervention session to read the entire conversation. After reading the sample conversation, the experimenter discussed the characteristics of the conversations with the participants (“In the conversation the friends asked each other questions, responded to their friend’s questions and talked about things that they both liked”). The participants were then instructed to have a text conversation with their peer, similar to the one in the guidebook. The experimenter also provided assistance as needed during the session such as if they had any questions for their friend and reminding them to read what their peer wrote before responding. After 10 min of conversing, if the participants were still texting, the experimenter told them that it was time to end their conversation. Criterion was met when the participants maintained an appropriate back and forth conversation to 100% accuracy on two separate sessions. Then fading was implemented.

#### Fading

Fading consisted of removing the guidebooks and no longer providing prompting. The participants were then given 10 min to text with each other per session. The fading criterion was set at 80% or above of correct conversational texting on two consecutive sessions.

#### Booster Sessions

If a participant’s percentage of appropriate texting regressed back to baseline levels during fading after a single session, two booster sessions of intervention were presented. Then, fading was again implemented across two more sessions.

#### Independent Weekly Texts

After fading, the dyads were instructed to have a conversation with their friend at least once a week independently. Specifically, the experimenter was no longer present while the children texted, but rather examined the conversation after the fact using the permanent product (i.e., pictures of the conversations). The experimenter instructed the parents to help the dyads identify a day each week when they would have a texting conversation. A screenshot of the texting conversation was taken each week by the parent, and the texting conversation was examined in terms of occurrence, appropriate beginning and end to the conversation, appropriate language, the length of the conversation, staying on topic, and both asking and responding to peer questions.

#### Generalization Texting Partner Probes

These generalization probes were implemented during baseline, following intervention fading, and at 1-month follow-up. Generalization probes involved the same conditions as in baseline except each participant texted another communication partner that they were likely to text in the future.

#### FaceTime® Probes

These probes also had the same conditions as in baseline, but instead of texting, the participant made a FaceTime® call to their friend. Because FaceTime® was not taught in this study, the steps to access FaceTime® were done by the experimenter or parent. Specifically, this probe accessed the ancillary effects texting with a peer has on the verbal conversation skills via the smart phone.

#### Follow-up

Follow-up data were collected 1 month following treatment. The conditions during follow-up were identical to baseline.

### Measures

#### Dependent Measures

Texting content included the use of greetings at the beginning and end of the conversation, appropriate language, staying on topic, appropriate length of texts, and both asking and responding to peer questions. The text conversations were scored by examining whether the conversation as a whole was contextually appropriate. A summary of the operational definitions for the dependent variables and examples is presented in Table 2.

**Table 2:** Dependent variables

Dependent measures	Operational definition	Example
The participant sent at least one text	The participant completed the text	1. Opened the text app. 2. Touched the new message or previous message buttons. 3. Typed in name. Clicked on name. 4. Clicked on message box. 5. Typed in a message. 6. Sent the message
Appropriate beginning to the conversation	The participant said some form of the word hello at the beginning of the conversation	“Hi,” “Hi _____,” “Hello,” “Hey,” etc.
Appropriate language	The participant did not talk about inappropriate topics	Participant does not send a text to his or her peer about their bathroom habits
Length of the conversation and individual texts	The participant sent at least five texts per conversation and no single text was more than 4 lines. No one word single texts three times in a row and no repeated texts.	Five different texts bubbles in a single color and 1–4 lines of text per each bubble. There were not text bubbles in a row that contained only single words (e.g., “yes,” “cool,” or “fine”). No participant sent two texts that were the same (e.g., “I like candy,” “I like candy”).
Staying on topic	The text message the participant sent was related or in some way referenced the texts preceding it	Text from peer: “My favorite sport is baseball!,” response from participant: “That’s cool! I like soccer!”
Asking questions	The participant asked his/her peer at least one question per conversation	“What’s your favorite sport?,” “Do you have any siblings?”
Responding to questions	The participant responded to at least one question of his or her peers per conversation	“I like basketball,” “I have three sisters”
Novel response	The participant’s texts differed from the texts presented in the sample conversations	“I really love science class,” “It was fun chatting”
Appropriate end to the conversation	The participant said some form of the word goodbye at the end of the conversation.	“Bye,” “See ya,” “See you later,” etc.

#### Novel Content

The operational definition of novel content was that the topic was not used in the same conversation and not used in more than two other conversations. The operational conversation was that the content had not been used in a prior conversation.

#### Ancillary Measure

In order to assess for potential collateral effects (Ledbetter-Cho et al., 2017), we evaluated how the impact of learning to text with a peer subsequently affected the content of verbal back and forth conversations between peers when communicating over FaceTime® (see Table 3 for operational definitions and examples). The conversational content was scored similarly to that of the dependent variables.

**Table 3:** Ancillary variable

FaceTime® measures	Operational definition	Example
Appropriate beginning to the conversation	The participant said some form of the word hello at the beginning of the conversation	“Hi,” “Hi _____,” “Hello,” “Hey,” etc.
Appropriate language	The participant did not talk about inappropriate topics	Participant did not ask peer about his or her bathroom habits
Staying on topic	Each statement said by a participant was related or in some way referenced preceding statements or questions	Friend: “My favorite sport is baseball!” Participant: “That’s cool! I like soccer!”
Asking questions	The participant asked his/her peer at least one question per conversation	“What’s your favorite sport?” “Do you have any siblings?”
Responding to questions	The participant responded to at least one question of his or her peers per conversation	“I like basketball” “I have three sisters”
Time spent speaking	No single participant talked for more than 20 s straight (no monologues) No one word responses three times in a row	Participant did not speak for 20 seconds without stopping to give their friend a turn Participant did not say “good,” “yes,” or “fine” in three consecutive responses
Appropriate end to the conversation	Participant said some form of the word goodbye at the end of the conversation	“Bye,” “See ya,” “See you later,” etc.

### Data Analyses

#### Interrater Reliability and Procedural Fidelity

The primary research observer and one secondary observer were trained on how to score the texting conversations for content and how to score the ancillary measures. To assist with scoring, checklists were provided that contained the observational definitions. The secondary observer reviewed the permanent products of 33% of the texting conversations across conditions, along with 33% of the videotapes of baseline, intervention training, generalization texting partner probes, FaceTime® probes, and follow-up sessions for each participant. The primary and secondary observers then compared scores to each other to determine interobserver agreement. If scorers disagreed, they re-watched the videotapes and re-examined the permanent product of that session to resolve discrepancies. Interrater reliability was high across both participants and phases of the study, ranging from 88 to 100%. A summary of interrater reliability across participants can be seen below in Table 4.

**Table 4:** Interrater reliability

	Texting steps	Texting content	FaceTime® content	Gen-probe Steps	Gen-probe content
Bennett	100%	98%	88%	93%	100%
Milo	100%	93%	88%	100%	95%
Anna	100%	95%	88%	100%	90%
Veronica	100%	95%	94%	100%	90%

Additionally, two observers who did not participate in the texting intervention assessed procedural integrity in 33% of the sessions across conditions and participants. This was done using the videotapes of the baseline, intervention training, generalization texting partner probes, FaceTime® probes, and follow-up sessions for each participant. The observers received training on how procedures were implemented and were each given a check sheet to use when scoring the presence and absence of each step in the procedure across sessions and participants. Procedural fidelity for all participants ranged from 94 to 100% on average. Mean procedural fidelity for Bennett was 95%, Milo = 95%, Anna = 94%, Veronica = 94%, and Levi = 100%. The only errors involved the experimenter not ending a few of the sessions at exactly ten minutes.

## Results

During baseline, all four participants demonstrated basic texting skills (i.e., the steps required for sending and receiving a text). However, none of the participants demonstrated the use of appropriate conversational text content. Following intervention, all four participants texted appropriately with their peers. Specifically, all of the participants met the criterion for sending a text and producing 100% appropriate content, across two consecutive texting sessions. Generalization partner probes suggested that participants generalized to their texting partners (parents and siblings), scoring 80–100% following the intervention being fully faded. The participants also maintained their skills at 1-month follow-up. The ancillary measure of the percentage of appropriate content discussed through FaceTime® also increased for all participants in the probes following intervention. In addition, this ancillary variable, verbal content, continued to be present at high rates in the FaceTime® probes at 1 month following completion of the texting intervention. Importantly, varied and novel topics of conversation increased within the texting conversations and the FaceTime® probes.

### Dyad 1: Bennett and Milo

#### Bennett

**Fig. 2:**
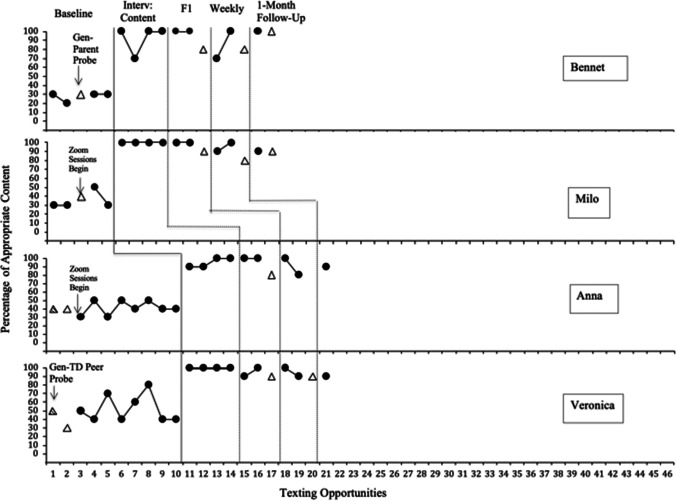
Percentage of appropriate text content for Bennett, Milo, Anna, and Veronica during baseline, intervention, content generalization partner probes, fading version 1, weekly texts, 1-month follow-up

In his four baseline sessions and one generalization partner probe (mother), Bennett demonstrated a low percentage of appropriate texting content (see Fig. 2, panel 1). During intervention, Bennett scored 100% and 70%, respectively, during his first two sessions and then completed his third and fourth intervention sessions by scoring 100% on both sessions. After reaching this criterion, the materials were fully faded. Bennett met fading criterion by scoring 100% on appropriate content during his two fading sessions. He demonstrated generalization of the skills across texting partners by scoring 80% on his generalization parent probe. During his two weekly independent texting sessions, he scored 70% and 100%, respectively. Bennett also demonstrated continued generalization of the skill to his mom, scoring 80% on the generalization texting probe following the weekly texts. In addition, Bennett maintained the skill 1 month following treatment and continued to generalize to his mother, by scoring 100% on both sessions.

#### Milo

During Milo’s four baseline sessions and one generalization partner probe (mother), he demonstrated a low percentage of appropriate texting content across sessions (see Fig. 2, panel 2). Milo’s percentage of appropriate text content was 40% during his baseline generalization partner probe (mother). After the texting content intervention was implemented, his percentage of appropriate texting content increased to 100% in all four intervention sessions. After fading, Milo continued to demonstrate 100% of appropriate text content across two consecutive sessions. He also demonstrated generalization from his peer to his mom by scoring 90% on his generalization partner probe. During the two weekly independent texting sessions, Milo continued to text appropriately at 90% and 100%, respectively. Milo also demonstrated a continued generalization of the skill across texting partners by scoring 80% on his generalization parent probe. Lastly, Milo demonstrated continued maintenance and generalization of the skill 1 month following treatment, scoring 90% on both the follow-up session and the generalization probe.

### Dyad 2: Anna and Veronica

#### Anna

During her eight baseline sessions and two generalization partner probes (typically developing peer and mom), Anna demonstrated a low percentage of appropriate texting content (see Fig. 2, panel 3) with her conversation partner as well as her generalization texting partner probes (mom and typically developing peer). Anna also met criterion within four intervention texting sessions. After the intervention was faded, Anna met and surpassed the fading criterion by continuing to demonstrate 100% of appropriate text content across two more sessions. Anna also generalized across texting partners by scoring 80% on her generalization partner probe (mother). While completing her two weekly text conversations, Anna continued to demonstrate skill acquisition by scoring 100% and 80%, respectively. In addition, Anna also showed maintenance of the skill by scoring 90% at 1-month follow-up.

#### Veronica

Across Veronica’s eight baseline sessions and two generalization probes, she produced a low variable rate of text content (see Fig. 2, panel 4). She scored 50% on her baseline generalization partner probe with a typically developing peer and 30% on her generalization partner probe with her mother. During her four texting intervention sessions, she produced 100% appropriate text content across sessions, reaching criterion. After the intervention was faded, she met fading criterion by producing 90% and 100% of appropriate content during two consecutive sessions. Veronica also generalized the skill across texting partners by scoring 90% on her parent probe. When completing the two weekly texting sessions, she maintained the skill by scoring 100% and 90%, respectively. Veronica also demonstrated maintenance of the skill by scoring 90% on her 1-month follow-up session.

### Novel Content

#### Bennett and Milo

Both children increased the amount of novel topics discussed (topics not discussed in the sample conversations or previous conversations) during baseline versus intervention. Specifically, they increased from two topics to seventeen topics. Specific topics discussed in baseline and during content intervention are listed in Table 5.

**Table 5:** Specific topics discussed by Bennett and Milo during baseline and intervention

Baseline	Content intervention
• Applications on the phone • Favorite foods	• Favorite countries • Favorite types of dinosaurs • Favorite videogames • Favorite states • School subjects • Favorite types of soda • Favorite foods • Television shows • Pets • Things they are allergic to • Favorite songs • Favorite ice cream flavors • Favorite pizza toppings • Favorite cities • Activities they want to do before they turn 18 • Rules they would make if they could be a parent • Favorite fastfood places

#### Anna and Veronica

This dyad’s number of novel topics increased from seven topics in baseline to ten topics during intervention (see Table 6 for the list of topics discussed). The topics discussed during baseline were mostly centered on topics of preoccupations.

**Table 6:** Specific topics discussed by Anna and Veronica during baseline and intervention

Baseline	Content intervention
• Specific television show • Game (on the phone) • Bruno Mars music • Trips • Boba • Blaze Pizza restaurant • Favorite pizza toppings	• Music • Applications on the phone • Trips • Apple products • What time they go to sleep at • How the day is going • Their thoughts on social distancing • Food • Activities they are doing • Weekend plans

### FaceTime® Probes

#### Bennett

**Fig. 3:**
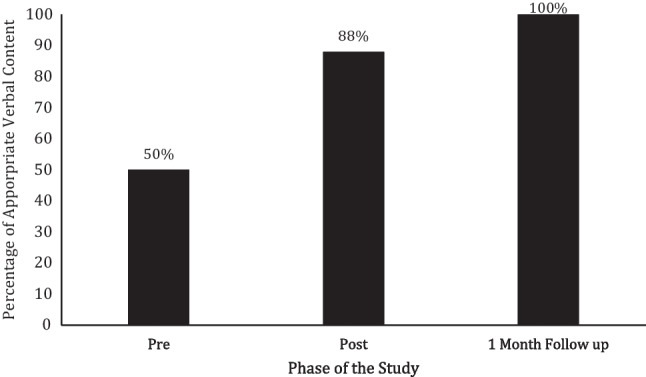
Bennett’s percentage of appropriate verbal content during FaceTime® probes taken prior to, directly after, and 1 month following the texting content intervention being faded

During his baseline FaceTime® probe, Bennett scored a 50% on appropriate verbal conversational content with his texting partner (see Fig. 3). Following the texting content intervention and fading, he scored an 88% on appropriate verbal conversational content. At 1-month follow-up, Bennett scored a 100% on appropriate verbal content.

#### Milo

**Fig. 4:**
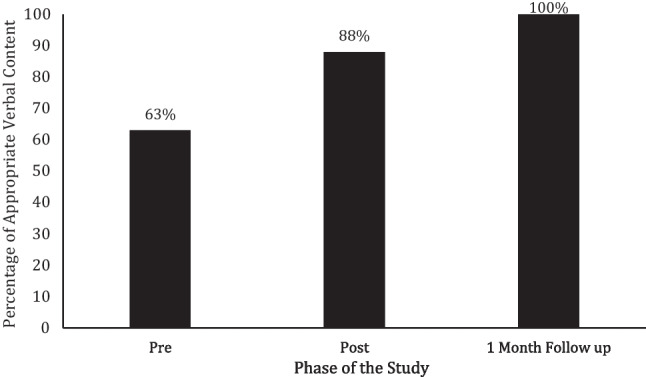
Milo’s percentage of appropriate verbal content during FaceTime® probes prior to, directly after, and 1 month following the texting content intervention being faded

In his baseline, FaceTime® probe, Milo’s percentage of appropriate verbal conversational content occurred was 63% (see Fig. 4). After the texting content intervention was presented and faded, he scored an 88% on percentage of appropriate verbal content during his FaceTime® probe with his texting partner. During Milo’s final FaceTime® probe, which was taken 1 month following the texting content intervention being faded, he scored a 100% on appropriate verbal content.

#### Anna

**Fig. 5:**
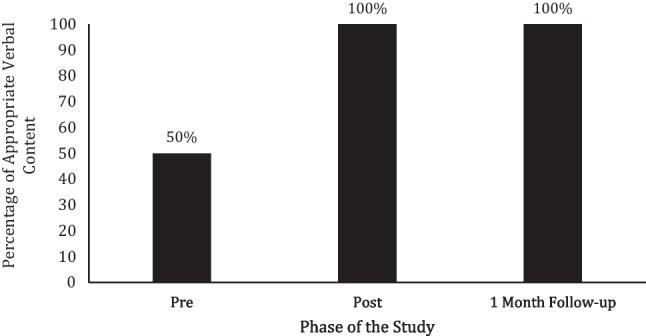
Anna’s percentage of appropriate verbal content during FaceTime® probes prior to and following the texting content intervention

Anna’s percentage of appropriate verbal content was 50% during her baseline FaceTime® probe with her texting partner Veronica (see Fig. 5). In the FaceTime® probe following the texting content intervention being faded, she scored a 100%. She also demonstrated 100% of appropriate verbal content on her FaceTime® probe taken at 1-month follow-up.

#### Veronica

**Fig. 6:**
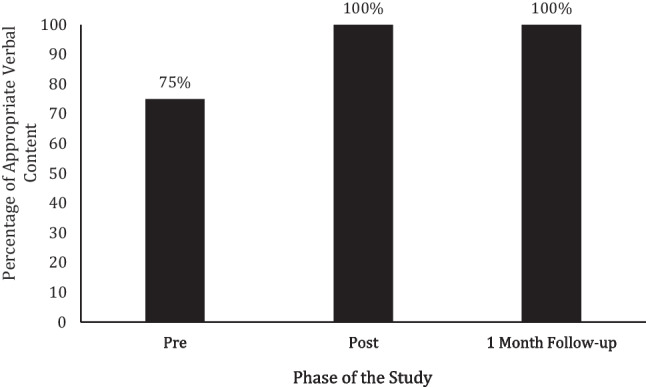
Veronica’s percentage of appropriate verbal content during FaceTime® probes prior to and following the texting content intervention

Veronica’s percentage of appropriate verbal content was 75% during her baseline FaceTime® probe (see Fig. 6). Following the texting content intervention being faded, she scored 100% during her FaceTime® probe as well as at 1-month follow-up.

## Discussion

This study experimentally examined text messaging as a potential avenue for social communication for autistic children. All four participants increased their appropriate text content after receiving a texting intervention. They generalized this skill across texting partners, and maintained the skill during the weekly independent texts and at the 1-month follow-up. The children increased the number of novel topics discussed, and the participants increased their appropriate conversational content during FaceTime® probes.

The purpose of the texting intervention was to increase appropriate content in text exchanges by the dyads of autistic children. During baseline, while the children did engage in some appropriate text content, much of their content was deemed inappropriate or based on “preoccupations with specific topics,” a hallmark characteristic of autistic children (APA, 2013). However, each dyad met criterion after only four consecutive training sessions.

One possible reason for the children’s rapid acquisition might be that the intervention was a visually oriented procedure. A guidebook was used with pictures of the text conversations on a smart phone. This was similar to Brodhead et al. (2019) who presented their visual scripts in a similar manner. Indeed, numerous studies have supported the use of visual programming for autistic children (e.g., Apple et al., 2005; Boudreau & Harvey, 2013; Charlop et al., 2008; Charlop et al., 2010; Grosberg & Charlop, 2017; Macpherson et al., 2015; Nikopoulos & Keenan, 2003; Sherer et al., 2001).

Additionally, a multiple exemplar training approach was used in which two different sample conversations were presented. Research has long hailed the benefits of multiple exemplar training (e.g., Brodhead et al., 2019; Charlop et al., 2008; LaFrance & Tarbox, 2019). Multiple exemplar training facilitates stimulus as well response generalization. An important finding in the present study was that all four participants generalized their texting conversational skills from their peer to their parent, and for one participant, to his sibling as well. This stimulus generalization boosts the significance of the present results and is consistent with prior research that includes multiple exemplars (Charlop-Christy & Kelso, 2003; LaFrance & Tarbox, 2019; Marzullo-Kerth et al., 2011; Pollard et al., 2012).

Fading all non-natural stimuli (i.e., guidebooks, prompting, and experimenter’s presence) may have been beneficial in promoting independent texting, which the children engaged in during the week. Consistent with previous research, fading out of such stimulus materials does support the independent production of behavior (e.g., Brown et al., 2008; Pollard et al., 2012). In the present study, the motivation for texting with both peers and family members (generalization partners) may have increased in value during the time of the study. Due to Covid-19, government issued lockdown, school virtual programming, and other environmental safety closures, social activities were severely curtailed for the participants. These public health requirements may have acted as an establishing operation (EO) that increased the value of communicating with friends and family members through text (Michael, 2000). Although this is an unfortunate way to highlight the value of the texting procedure, it may also be a new way of looking at social communication in other contexts also. For example, one of the dyads consisted of children who lived on opposite sides of the county and traffic, parental work schedules, and other logistics would make it difficult for the girls to have in-person play dates. Texting is now a way for these participants to continue their social communication and friendship.

Independent texting was an important aspect of the study. This occurred when the participants decided together that they wanted to text and at what point to end the conversation. Such as natural extension of this research was an important component of the study that may have helped promote maintenance and follow-up at 1 month. This component created a texting environment that the child could naturally experience in the future (Peterson, 2009).

The response generalization to the FaceTime® probes was another positive finding in the present study. One reason this transfer of content skills may have occurred could be due to the similarity of the content discussed over text and verbally (i.e., shared topics of interest, asking questions, responding to questions). Another potential explanation for this ancillary effect could be because following the text exchanges; the children began to learn more information about each other and strengthened their relationships, which in turn provided more content to discuss during the FaceTime® probes taken after the texting intervention and during follow-up.

The increase in the novel responses after the texting intervention might provide further support for the texting intervention. The incorporation of the multiple exemplars in the text conversations within the guidebooks during intervention might have a facilitative effect on the children’s social communication (e.g., Charlop et al., 2008).

### Limitations and Future Research

A limitation of this study was the number of participants. There were only four autistic children, comprising two dyads. Therefore, this study needs to be replicated with a larger participant pool of autistic children and teens. Another limitation is that the procedure needed to change for some participants in middle due to Covid shelter at home governmental orders.

The future of texting as a means of social communication for autistic children is particularly relevant in present times. The results of this study hold promise for non-Covid-19 conditions. Autistic children who live a distance away from each other can maintain social communication and friendship similarly as neurotypical children. Everyday logistics such as traffic, parental work schedules, after-school activities, and weather conditions may preclude social skill opportunities, which are important for autistic children. As well, those children who live in areas that are more rural can access peers more readily via text. Finally, it has also been argued that texting may have positive effects on both language and literacy skills (Crystal, 2008; Plester et al., 2009). Given the positive results of the present study of the FaceTime® probes, texting may promote face-to-face conversational skills as well.
